# Prognostic implications of organ-specific metastases in advanced gastric cancer: A retrospective observational analysis of the SEER database

**DOI:** 10.1097/MD.0000000000045570

**Published:** 2025-10-31

**Authors:** Guihua Liu, Junjun Gu, Jing Wang, Fang Li, Yi Shen, Zhimin Wang, Xianglong Tian

**Affiliations:** a Department of General Practice, Minhang District Hongqiao Community Health Service Center, Shanghai, China; b Department of Gastroenterology, Shanghai Eighth People’s Hospital, Shanghai, China; c Department of Physical Examination Center, Shanghai Armed Police Corps Hospital, Shanghai, China; d Department of General Practice, Zhongshan Community Health Service Center of Songjiang District, Shanghai, China; e Department of Emergency, RuiJin Hospital Lu Wan Branch, Shanghai Jiaotong University School of Medicine, Shanghai, China; f Department of Gastroenterology, Central Hospital of Sakya County, Tibet, China.

**Keywords:** gastric cancer, metastases characteristics, nomogram, prognosis, SEER

## Abstract

Advanced gastric cancer (GC) remains a significant global health burden with poor prognosis. Understanding organ-specific metastatic patterns and their prognostic implications is critical for optimizing patient management. This study leverages the Surveillance Epidemiology and End Results database to comprehensively analyze metastatic patterns in GC and develop a robust prognostic model. We analyzed data from 10,842 GC patients diagnosed between 2010 and 2014, focusing on metastases to the liver, lungs, bones, and brain. Metastatic patterns, prognostic outcomes, and risk factors were evaluated using multivariable logistic and Cox regression analyses. A nomogram was developed to predict overall survival. Liver metastases were the most common (40.5%), followed by lung (13.5%), bone (11.0%), and brain (1.7%). Dual-organ metastasis most frequently involved the liver and lungs. Patients with isolated liver metastases had a relatively better prognosis (hazard ratio = 1.29, 95% confidence interval = 1.23–1.36, *P* < .0001), while those with isolated bone metastases had the poorest outcomes (hazard ratio = 1.99, 95% confidence interval = 1.63–1.96, *P* < .0001). Prognosis was uniformly poor for patients with metastases to 2 or more organs. Key risk factors included male sex, older age, and poorly differentiated tumors. A nomogram incorporating these factors demonstrated strong predictive accuracy. This study provides a comprehensive analysis of organ-specific metastatic patterns in GC, highlighting the prognostic significance of metastatic sites. The developed nomogram offers a practical tool for clinicians to predict survival outcomes and tailor treatment strategies for advanced GC patients.

## 1. Introduction

Gastric cancer (GC) ranks as the fifth most prevalent cancer globally and stands as the fourth leading cause of cancer-related deaths, contributing to ~770,000 fatalities annually.^[1]^ Despite substantial advancements in cancer treatment, the prognosis for GC remains disheartening, with a 5-year survival rate hovering around 20% in most regions worldwide.^[2,3]^

Metastasis is a prevalent feature of GC and stands as a significant contributor to treatment failure and unfavorable outcomes.^[4]^ While early-stage GC patients are typically curable, most GC cases are diagnosed at the intermediate and advanced stages.^[5,6]^ Reports indicate a grimmer prognosis with the spread of GC, featuring a 5-year survival rate of approximately 20%.^[7,8]^ Given the substantial number of patients with advanced GC, standardized management for this subgroup is indispensable. It is imperative to comprehend the metastatic characteristics and discern the risk factors associated with organ metastases to enhance the management and prognosis of advanced GC patients.

Earlier research has reported that metastases frequently manifest in the liver and lungs, whereas occurrences in bones and the brain are relatively rare in GC.^[9,10]^ However, the detailed examination of metastatic patterns and their influence on survival outcomes in GC has not been extensively explored. Therefore, we intend to delve into the characteristics of metastases in GC, discern the risk factors linked with organ metastases, and scrutinize survival disparities based on metastatic sites.

To fulfill these objectives, we leveraged the Surveillance Epidemiology and End Results (SEER) databases, a valuable resource providing comprehensive information on the incidence of organ metastases in patients with GC. The primary aim of this study is to provide valuable insights to clinicians for improving the management of patients with advanced GC.

## 2. Materials and methods

This study harnessed data from the SEER databases, presenting insights into GC patients diagnosed between 2010 and 2014. The inclusion criteria encompassed GC patients with meticulously documented organ metastases information, yielding a dataset of 10,842 samples that met the analysis prerequisites. Retrieval of GC patient data was conducted through the SEER*Stat software (version 8.3.5). The specific database employed was the SEER Program (www.seer.cancer.gov) Research Data (1973–2014), National Cancer Institute, DCCPS, Surveillance Research Program, released in April 2017, based on the November 2016 submission.

This study used publicly available, de-identified data from the SEER database. As no patient personal identifying information (e.g., names, addresses, medical record numbers) was accessed, used, or analyzed, formal ethical approval from an ethics committee was unnecessary. Informed consent from patients was also not required, given the lack of personal identifying information used.

For missing data handling, a complete case analysis approach was adopted. If any key variable (e.g., pathological grading, marital status, economic income) required for statistical analyses was missing in a sample, the sample was excluded from the corresponding analysis.

In our study, seer data provided information on liver, lungs, bones and brain metastases of GC. Demographic and clinicopathological characteristics of the patients were summarized, and the χ^2^ test was employed to compare categorical variables across diverse groups. The study comprehensively examined the frequency of metastases in different organs and explored potential associations between metastases in various anatomical sites. The assessment of overall survival (OS) involved the application of the Kaplan–Meier estimator method, with survival curves compared using the log-rank test. To discern factors associated with organ metastases, Multivariable logistic regression analysis was taken, including variables such as gender, age, race, ethnic origin, pathological grading, tumor stage, marital status, education level, and economic income. For the evaluation of potential prognostic factors, Multivariate Cox proportional regression analysis was applied, yielding hazard ratios (HR) and their corresponding 95% confidence intervals (CI). A nomogram, grounded on GC metastases, was built to predict OS. The nomogram’s accuracy was assessed through identification and calibration methods, along with decision curve analysis.

The statistical analyses were conducted using R software (version 4.2.0). A *P*-value of <.05 on both sides was considered statistically significant.

## 3. Results

### 3.1. The demographic and clinicopathological characteristics

Patients diagnosed with GC between 2010 and 2014 were extracted from the SEER databases, which comprehensively cataloged metastatic information, encompassing liver, lungs, bones, and brain metastases. Following the exclusion of samples with unspecified organ metastasis data, our study identified a total of 10,842 samples. Among these, 5030 cases exhibited organ metastases, while 5812 cases (53.6%) did not manifest organ metastases in the aforementioned sites. Table 1 illustrated the association between the clinicopathological features of GC patients and overall organ metastases, while Table S1, Supplemental Digital Content, https://links.lww.com/MD/Q488 provided a detailed breakdown of the association with individual organ metastases.

**Table 1:** The baseline demographic and clinical characteristics for GC in our study.

	With organ metastasis N = 5812 (53.6%)	without organ metastasis N = 5030 (46.4%)	χ^2^ test
Sex			
Female	1775 (46.6%)	2033 (53.4%)	<0.0001
Male	4037 (57.4%)	2997 (42.6%)	
Race			
American Indian/Alaska Native	54 (51.9%)	50 (48.1%)	<0.0001
Asian or Pacific Islander	633 (47.2%)	707 (52.8%)	
Black	852 (58.3%)	610 (41.7%)	
White	4251 (53.8%)	3644 (46.2%)	
Age			
Older than 65 yr	3115 (58.2%)	2236 (41.8%)	<0.0001
Younger than 65 yr	2697 (49.1%)	2794 (50.9%)	
Ethnic origin			
Non_Spanish_Hispanic_Latino	4851 (55.8%)	3841 (44.2%)	<0.0001
Spanish_Hispanic_Latino	961 (44.7%)	1189 (55.3%)	
Pathological grading			
Well differentiated; Grade I	1257 (64.3%)	699 (35.7%)	<0.0001
Moderately differentiated; Grade II	2834 (48.1%)	3058 (51.9%)	
Poorly differentiated; Grade III	115 (53.5%)	100 (46.5%)	
Undifferentiated; anaplastic; Grade IV	1470 (57.6%)	1084 (42.4%)	
Unknown	136 (60.4%)	89 (39.6%)	
Marital status			
Married (including common law)	3278 (52.9%)	2915 (47.1%)	0.0057
Separated or divorced	613 (56.3%)	476 (43.7%)	
Single (never married)	915 (51.8%)	852 (48.2%)	
Widowed	724 (57.0%)	546 (43.0%)	
Median household income (2008–2012)*			
Q1	1503 (54.6%)	1249 (45.4%)	0.0834
Q2	1438 (51.6%)	1347 (48.4%)	
Q3	1403 (54.7%)	1163 (45.3%)	
Q4	1468 (53.6%)	1271 (46.4%)	
High school education (2008–2012)*			
Q1	1067 (53.6%)	923 (46.4%)	0.0124
Q2	1257 (55.6%)	1003 (44.4%)	
Q3	1442 (54.7%)	1192 (45.3%)	
Q4	2046 (51.7%)	1912 (48.3%)	
OS_status			
Alive	1095 (44.7%)	1354 (55.3%)	<0.0001
Dead	4717 (56.2%)	3676 (43.8%)	

* Measure of educational level or economic income for each patient’s area of residence is from 2012 American Community Survey data, and it is categorized into equally proportioned quartiles.

Among the patients with organ metastases, males accounted for a higher proportion (57.4%). In terms of demographics, black individuals (58.3%) and non-Hispanic/Latino individuals (55.8%) showed a greater susceptibility to developing organ metastases. Marital status also played a role, as separated, divorced, or widowed individuals had a higher incidence of GC metastases. Intriguingly, there was no statistically significant association between the occurrence of GC metastases and the economic development of the region. As expected, advanced age and poorer pathological grading were strongly correlated with an increased risk of organ metastases.

### 3.2. Frequency and characteristics of organ metastases

**Figure 1.**
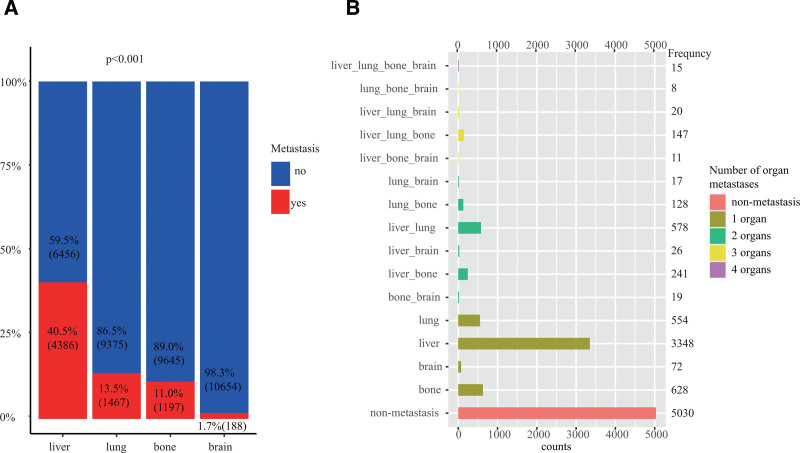
Bar plots indicate the distribution of gastric cancer (GC) metastases to different organs and by the number of affected organs. (A) Stacked bars show the frequency (n) and proportion (%) of metastases to 4 target organs (liver, lung, bone, brain) in 10,842 GC patients. (B) The bar chart shows the frequency of metastasis in GC patients based on the number of affected organs: non-metastasis, 1-organ metastasis, 2-organ metastasis, 3-organ metastasis, and 4-organ metastasis.

Upon preliminary analysis, as shown in Figure 1A, liver metastases emerged as the most prevalent, constituting 40.5% (4396 cases) of the total samples. Lungs metastases ranked the second position with a frequency of 13.5% (1464 cases), followed by bones metastases at 11.0% (1193 cases). Brain metastases exhibited the lowest frequency, accounting for merely 1.7% of the total sample (185 cases). Subsequent analysis was undertaken based on the number of organs affected. As depicted in Figure 1B, isolated liver metastases exhibited the highest frequency with 3348 cases, succeeded by bones metastases with 628 cases, and lungs metastases with 554 cases. Brain metastases recorded the lowest frequency with a mere 72 cases. In instances where GC metastases involved 2 organs, simultaneous liver and lungs metastases prevailed as the most frequent scenario (578 cases). Conversely, lungs and brain co-metastases displayed the lowest frequency with only 17 cases. In cases where 3 organs were implicated in GC metastases, the simultaneous involvement of the liver, lung, and bone exhibited the highest frequency (147 cases). Other metastasis patterns were relatively rare, with the lowest frequency observed in simultaneous metastases in the lungs, bones, and brain, accounting for only 8 cases. Notably, there were 18 cases characterized by simultaneous metastases in the liver, lung, bone, and brain. These comprehensive data afford valuable insights into the frequency of organ metastases across different organs and their various combinations.

**Figure 2.**
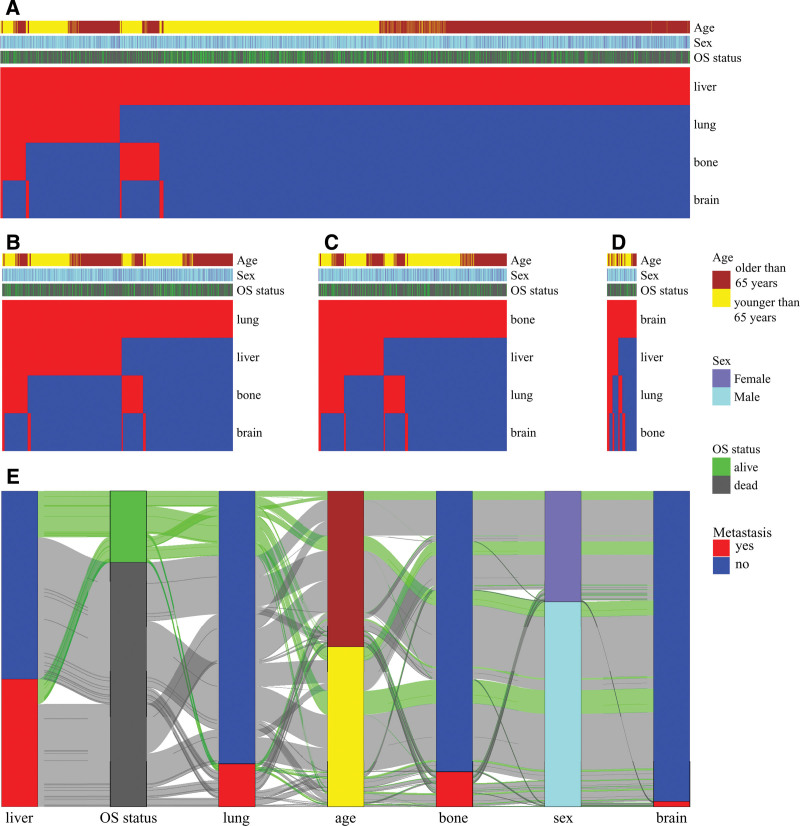
Heat maps and a Sankey diagram illustrate relationships between organ-specific metastases and clinical characteristics (sex, overall survival [OS] status, age at diagnosis) in GC patients. Heat maps show the distribution of these clinical characteristics and their association with single-organ metastases: (A) liver metastasis, (B) lung metastasis, (C) bone metastasis, and (D) brain metastasis. (E) Sankey diagram: Bars represent variables (organ metastases, sex, OS status, age), the gray and green lines between the bars reflect the congruencies and differences between the different variables. and connection line width reflects the magnitude of congruence between variables (wider lines indicate stronger associations). GC = gastric cancer.

After the metastatic analysis, we delved into the correlation between organ metastases and scrutinized their associations with gender, age, and survival status. Figure 2A portrayed a heightened proportion of patients characterized by isolated liver metastases. Following the onset of liver metastases in GC, approximately one-quarter of patients subsequently developed lungs or bones metastases, although the occurrence of simultaneous lungs and bones metastases remained relatively low. Notably, patients with combined bone and liver metastases exhibited a higher risk of mortality compared to those with combined lung and liver metastases. Figure 2B depicted that fewer than half of the patients presented with isolated lungs metastases, with the majority showcasing metastases to other organs. Additionally, the prevalence of liver metastases surpassed that of bone metastases. Among patients with bones metastases (Fig. 2C), the risk of developing liver and lungs metastases was comparable. In Figure 2D, brain metastases emerged as the least likely, and when they did occur, approximately half of the patients already harbored metastases in the liver, lung, and/or bone. Intriguingly, isolated GC brain metastases were more prevalent in elderly patients. The relationships between liver metastases, lungs metastases, bones metastases, and brain metastases in GC with patient age, gender, and survival status were detailed in the Sankey diagram below (Fig. 2E).

### 3.3. Prognostic analysis based on different organ metastases in GC

**Figure 3.**
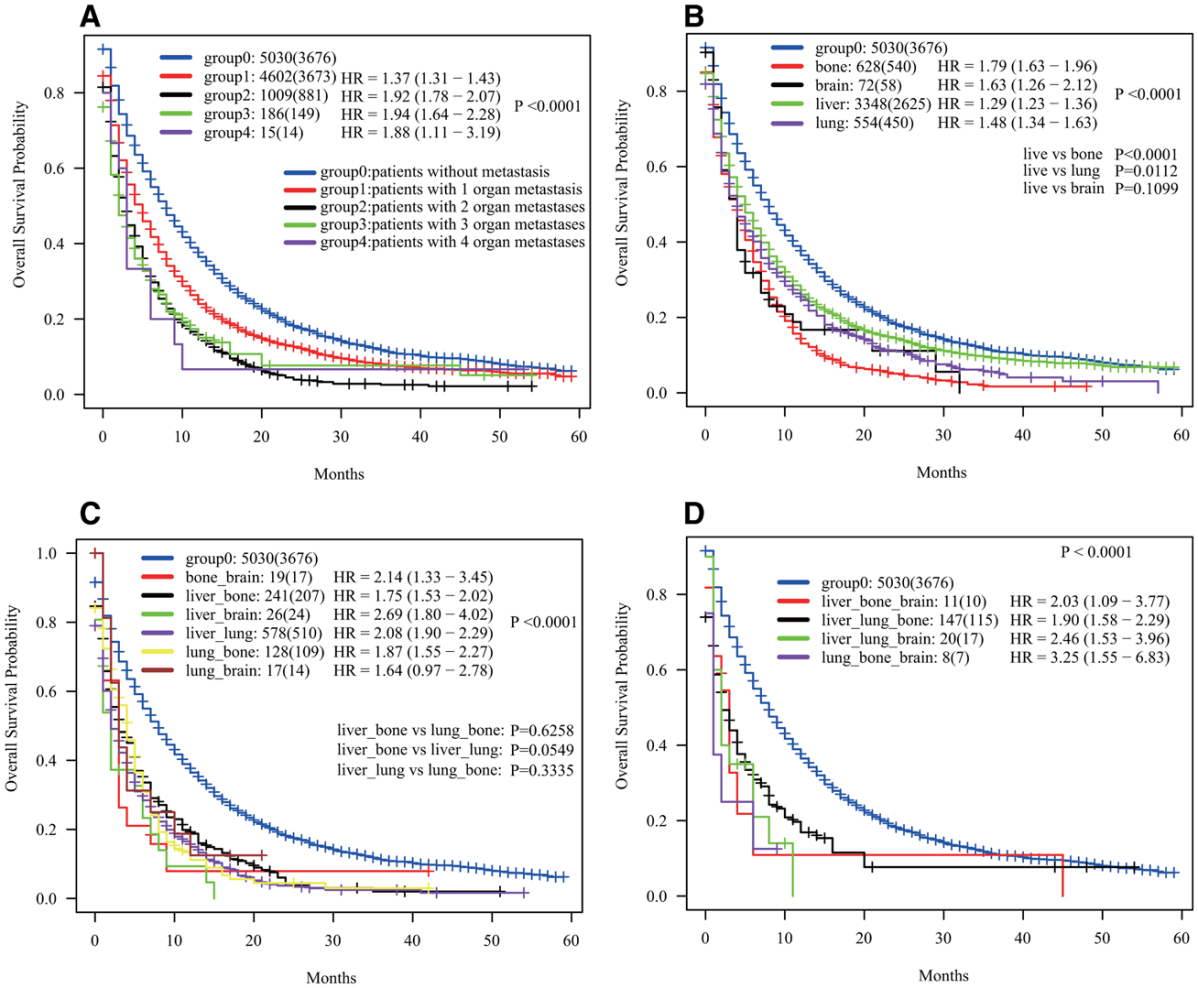
Kaplan–Meier survival analyses for advanced GC patients stratified by the number and site of organ metastases. (A) OS by number of metastatic organs: non-metastasis, 1-organ metastasis, 2-organ metastasis, 3-organ metastasis, and 4-organ metastasis. (B) OS by single metastatic organ: isolated liver metastasis, isolated lung metastasis, isolated bone metastasis and isolated brain metastasis. (C) OS by 2-organ metastases: liver-bone, liver-lung, lung-bone; no statistical differences between pairs (excluded 2-organ groups with brain metastasis, n ≈ 20, due to small sample size). (D) OS by 3-organ metastases: groups with concurrent brain metastasis had the worst prognosis (excluded for further discussion due to small sample size).

We further analyzed the prognosis differences of GC patients with different organ metastases using the Kaplan–Meier estimator method. Firstly, based on the number of affected organs, we divided the patients into 4 groups. The analysis results depicted in Figure 3A align with our expectations. Patients without organ metastases exhibited the most favorable outcomes, followed by those with only one organ affected. Comparatively, patients with a singular organ affected experienced a shorter OS (HR = 1.37, 95% CI = 1.31–1.43, *P* < .0001) in contrast to those without organ metastases. Patients with 2 or 3 organ metastases displayed even higher HR values, measuring 1.92 and 1.94, respectively. It was noteworthy that the survival curves for patients with 2 or more organs affected almost overlapped, signifying that once GC metastasized to 2 or more organs, the prognosis became exceedingly poor. Due to the limited number of GC patients with 4 organ metastases (only 15 cases), further discussion was not conducted to avert potential significant bias.

Subsequently, we explored the prognosis differences among patients with only one organ metastasis. As illustrated in Figure 3B, patients with isolated liver metastases demonstrated a relatively better prognosis (HR = 1.29, 95% CI = 1.23–1.36, *P* < .0001), while those with isolated bones metastases had the worst prognosis (HR = 1.99, 95% CI = 1.63–1.96, *P* < .0001). Detailed pairwise comparisons underscored statistically significant differences between isolated liver metastases and isolated bones metastases, as well as between isolated liver metastases and isolated lungs metastases.

Among patients with 2 organ metastases, the number of cases featuring combined brain metastases was relatively small (around 20 cases), leading us to forego further analysis and discussion due to potential bias. HR values for liver & bones metastases, liver & lungs metastases, and lungs & bones metastases were 1.75, 2.08, and 1.87, respectively. Importantly, the lack of statistical significance in these pairs suggested that regardless of the specific organs involved, once GC metastasized to affect 2 organs, the patient’s prognosis was bleak (Fig. 3C). Figure 3D demonstrated the prognosis disparities among patients with 3 organ metastases. The outcomes indicated that patients with combined brain metastases faced the most adverse prognosis. Similarly, given the limited number of patients with combined brain metastases, further discussion was withheld to prevent potential bias.

### 3.4. Identification of risk factors associated with metastases in GC

**Figure 4.**
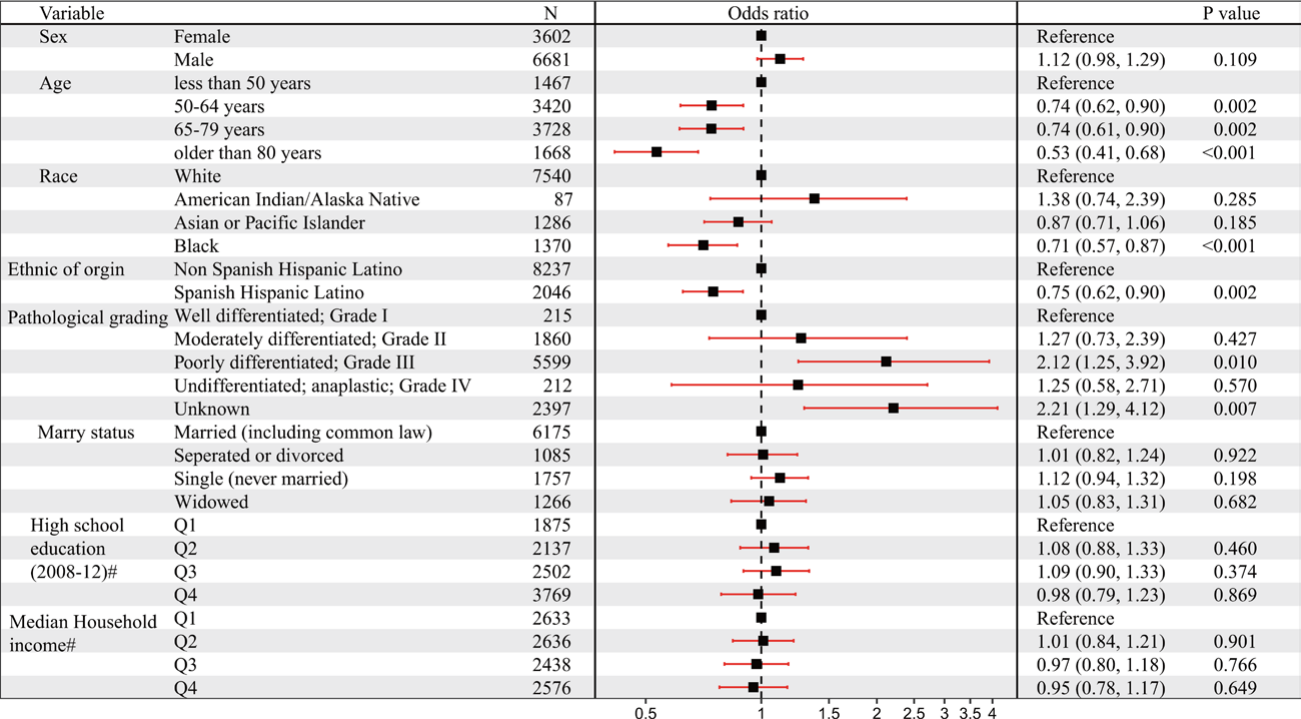
Identification of the risk factors associated with bone metastasis in advanced GC. Forest plot of Multivariable Logistic analyses of the risk of bone metastasis in GC patients adjusted for sex, age, race, ethnic origin, pathological grading, marital status, education level, and economic income. The black squares on the transverse lines represent the hazard ratio (HR), and the transverse lines represent 95% confidence intervals. # Measure of educational level or economic income for each patient’s area of residence is from 2012 American Community Survey data, and it is categorized into equally proportioned quartiles. GC = gastric cancer.

Significant differences in prognosis were evident among GC patients with different sites of metastases, with the worst prognosis associated with bones metastases and relatively better outcomes for liver metastases. Analyzing the risk factors for GC metastases, a multivariable logistic regression model in Figure 4 revealed several significant associations when adjusting for other factors. The risk of bones metastases escalated in patients with poorly differentiated (Grade III) GC (OR = 2.12; CI = 1.25–3.92; *P* < .001). Conversely, the risk of bones metastases was relatively lower in patients of Black race (OR = 0.71; CI = 0.57–0.87; *P* < .001), of Spanish Hispanic Latino ethnicity (OR = 0.75; CI = 0.62–0.90; *P* = .002), and aged over 50, especially in patients over 80 years old (OR = 0.53; CI = 0.41–0.65; *P* < .001). In contrast to bones metastases, poorly differentiated (Grade III) pathological grading acted as a protective factor for liver metastases (OR = 0.56; CI = 0.43–0.74; *P* < .001). Similarly, unlike bones metastases, the risk of liver metastases increased in patients aged over 50, with a pronounced rise in patients over 80 years old (OR = 2.07; CI = 1.76–2.41; *P* < .001). Black ethnicity was a protective factor for GC bones metastases but increased the risk of liver metastases (OR = 1.35; CI = 1.19–1.53; *P* < .001). Moreover, sex was associated with the risk of liver metastases, with male patients having a higher risk (OR = 1.52; CI = 1.39–1.66; *P* < .001). Further analysis revealed that Asian or Pacific Islander (OR = 0.76; CI = 0.66–0.86; *P* < .001) and Spanish Hispanic Latino (OR = 0.72; CI = 0.64–0.81; *P* < .001) populations had a relatively lower risk of GC liver metastases (see Fig. S1, Supplemental Digital Content, https://links.lww.com/MD/Q489). Additional analysis explored risk factors for GC lungs metastases and brain metastases. The results revealed that male patients (HR = 1.23; CI = 1.08–1.40; *P* = .001) and patients in a wisdom status (OR = 1.33; CI = 1.10–1.60; *P* = .003) were more likely to develop lungs metastases, while the patients of Asian or Pacific Islander (OR = 0.77; CI = 0.63–0.93; *P* = .007), Black ethnicity (OR = 0.76; CI = 0.23–0.78; *P* = .004), Spanish Hispanic Latino (HR = 0.77; CI = 0.65–0.90; *P* = .002), poorly differentiated Grade III (HR = 0.67; CI = 0.48–0.98; *P* = .032), and undifferentiated anaplastic Grade IV (OR = 0.43; CI = 0.63–0.91; *P* = .006) had a lower risk of lungs metastases (see Fig. S2, Supplemental Digital Content, https://links.lww.com/MD/Q489). Patients aged over 80 (OR = 0.37; CI = 0.18–0.72; *P* = .004), of Black ethnicity (OR = 0.47; CI = 0.27–0.79; *P* = .007), Spanish Hispanic Latino ethnicity (OR = 0.60; CI = 0.38–0.93; *P* = .028), and those with poorly differentiated Grade III (OR = 0.60; CI = 0.41–0.89; *P* = .009) had a lower risk of brain metastases (see Fig. S3, Supplemental Digital Content, https://links.lww.com/MD/Q489).

### 3.5. Analysis of prognostic factors using multivariable Cox regression

**Figure 5.**
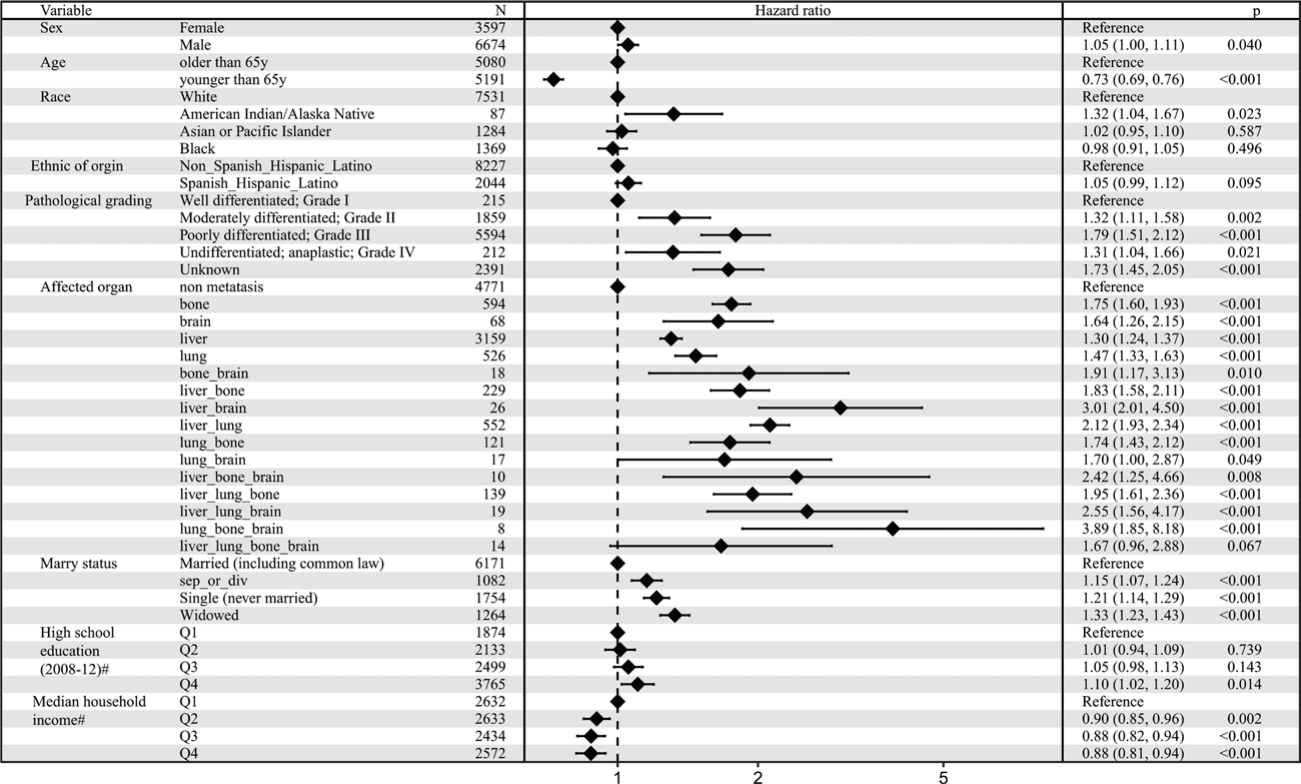
Identification of the prognostic factors in advanced GC. Forest plot of Multivariable Cox analyses in GC patients with no more than one organ metastasis adjusted for sex, age, race, ethnic origin, pathological grading, organ metastases, marital status, education level, and economic income. The black rhomboids on the transverse lines represent the hazard ratio (HR), and the transverse lines represent 95% confidence intervals. # Measure of educational level or economic income for each patient’s area of residence is from 2012 American Community Survey data, and it is categorized into equally proportioned quartiles. GC = gastric cancer.

To identify variables significantly impacting the prognosis of GC patients, multivariable Cox regression analysis was deployed, incorporating all relevant covariates. Figure 5 succinctly illustrated the covariates significantly associated with the OS of GC patients after adjusting for other factors such as sex, age, race, and pathological grading etc. The analysis revealed that the patient’s risk escalated if they were male, of American Indian/Alaska Native ethnicity, possess poorly differentiated tumors, and exhibit metastases to organs such as the liver, bones, lungs, and brain. Similarly, marital status played a pivotal role, with divorced, single, or widowed individuals facing an elevated risk. Contrastingly, younger patients and those with higher family income exhibited a relatively lower risk, as depicted in Figure 5.

### 3.6. Construction of nomogram based on GC metastases

To enhance the predictability of patient prognosis, we employed a randomized 3:2 patient sampling strategy, creating a training set and a validation set. Utilizing Cox regression analysis, we constructed a prognostic model in the training set, subsequently evaluating its predictive efficacy in the validation set. The Cox regression analysis underscored the significance of various factors including liver metastases, lungs metastases, bones metastases, brain metastases, tumor pathology grade, as well as patient age, marital status, and income in influencing the prognosis of GC patients.

**Figure 6.**
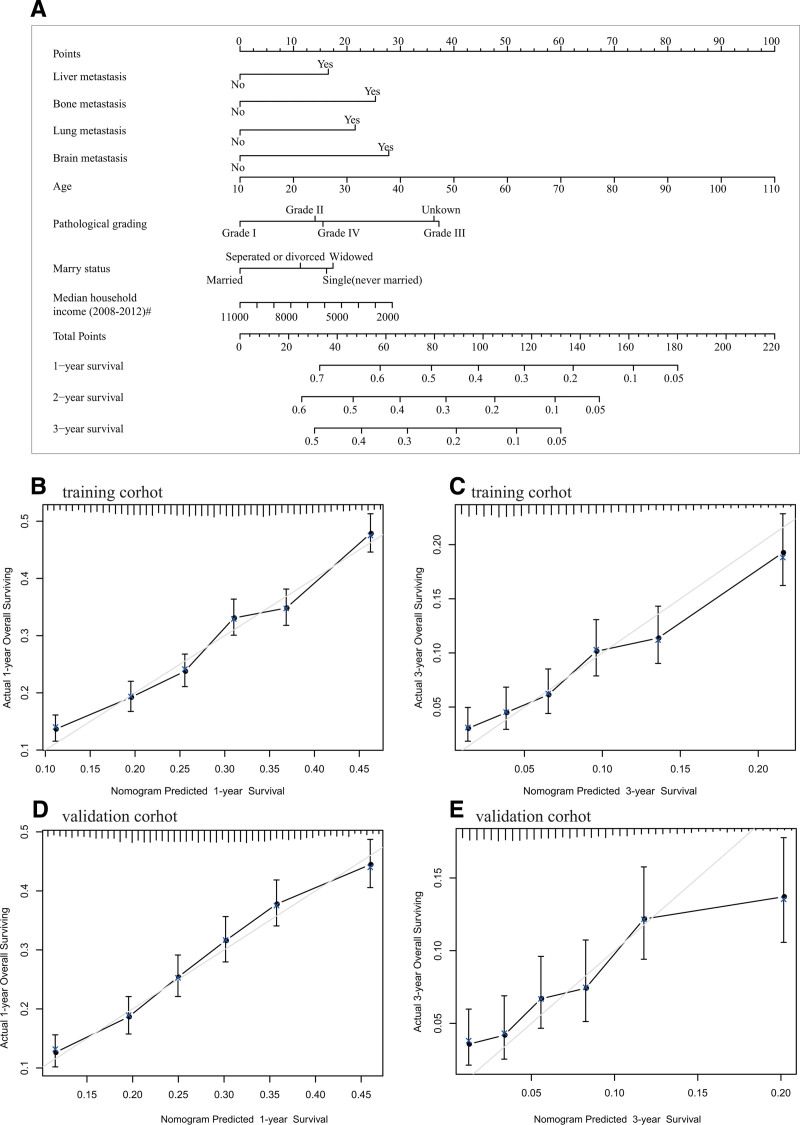
Nomogram for predicting 1-year, 2-year, and 3-year OS in advanced GC patients. (A) Nomogram: Each variable (liver/lung/bone/brain metastasis, tumor pathological grade, age, marital status, income) is assigned a weighted score; total score corresponds to predicted 1/2/3-year OS probability. (B–E) Calibration plots: X-axis indicates nomogram-predicted OS probability, Y-axis indicates actual OS probability (45° line = perfect prediction). (B) 1-year OS (training set), (C) 3-year OS (training set), (D) 1-year OS (validation set), (E) 3-year OS (validation set); line segments are closely aligned with the 45° line, indicating good calibration. GC = gastric cancer, OS = survival survival.

In a bid to facilitate clinical decision-making and prognostication, we developed a nomogram, assigning weighted scores to each parameter for predicting the 1-year, 2-year, and 3-year OS of GC patients (see Fig. 6A). The accuracy of the nomogram was rigorously assessed using identification and calibration methods, yielding a C-index of 0.629 in the training set and 0.627 in the validation set. Calibration plots in Figure 6B–E depict line segments closely aligned with the 45°-line, indicative of robust prediction performance, particularly for the 1-year prognosis. Further evaluation of the nomogram’s net benefit via Decision Curve Analysis revealed its potential to benefit GC patients, demonstrating reliable clinical utility (see Fig. S4, Supplemental Digital Content, https://links.lww.com/MD/Q489).

## 4. Discussion

This study aimed to explore the metastatic characteristics in GC, delineate the risk factors intricately associated with organ metastases, scrutinize the survival disparities contingent upon metastatic sites, and ultimately construct a clinically beneficial prognostic predictive model for GC patients. Our investigative efforts yielded valuable insights into the occurrence and patterns of organ metastases in GC. A crucial point necessitating clarification was the context of our analysis, which was specifically tailored for patients with advanced GC. Consequently, the observed proportion of organ metastases in all GC patients should logically be lower than the results extrapolated from our study. For instance, our findings indicated that the prevalence of bones metastases in patients with advanced GC was 11%. In contrast, reports revealed that the risk of bone metastasis in GC was comparatively lower, hovering around 4%.^[11,12]^

Our analysis of the SEER databases revealed that the liver was the predominant site of metastases in GC, closely trailed by the lung and bones, with brain metastases exhibiting the lowest occurrence rate. These outcomes resonate harmoniously with prior studies that consistently identify liver metastases as the most prevalent manifestation in GC.^[13]^ This notable predominance of liver metastases may be rationalized by the anatomical proximity of the liver to the stomach, thereby facilitating a more accessible conduit for the dissemination of cancer cells. The corroboration of our findings with established literature further bolsters the reliability of our study outcomes. Importantly, given that liver metastases account for the highest proportion of organ-specific metastases in our advanced GC cohort (40.5%), and isolated liver metastases were associated with a relatively better prognosis, exploring clinically actionable interventions for this subgroup becomes particularly meaningful. A population-based analysis focusing on GC with liver metastasis demonstrated that multimodal chemotherapy, including palliative chemotherapy and postoperative chemotherapy, was associated with improved overall survival compared to preoperative chemotherapy alone.^[14]^ This finding, combined with our observation of favorable prognosis in isolated liver metastasis, suggests that clinical management of advanced GC patients with isolated liver metastasis could prioritize the evaluation of multimodal chemotherapy eligibility, which may further enhance survival benefits for this specific subgroup.

Our scrutiny of the data unveiled a noteworthy trend in advanced GC patients, where liver metastases frequently co-occurred with lung and/or bones metastases. This proclivity towards cancer cells spreading to multiple organs suggested a complex and interconnected pattern of metastatic dissemination in advanced GC cases. Conversely, isolated lungs metastases were less prevalent, with a substantial proportion of these cases concurrently manifesting liver metastases. This implies that lungs metastases in GC often unfold in tandem with liver involvement. In the cohort with bones metastases, the risk of simultaneous liver and lungs metastases exhibited a comparable frequency. While brain metastases proved to be the least common, when it did occur, approximately half of the patients already harbored concurrent liver, lungs, and bones metastases. A finding that aligns with the insights from a propensity score-matched study on GC with brain metastasis, which emphasized the rarity of isolated brain metastasis and its tendency to occur alongside multi-organ involvement in advanced GC.^[15]^ Moreover, this study further explored imaging features of GC brain metastasis, which provides critical clinical references for the early detection of brain metastasis. Given that our study identified brain metastasis as a marker of poor prognosis, integrating the imaging characteristics from this study into clinical practice could facilitate timely diagnosis of brain metastasis, addressing the potential underdiagnosis issue similar to that observed in bone metastasis. These findings underscore the paramount importance of contemplating the potential for multi-organ involvement in advanced GC patients, thereby informing a comprehensive and nuanced clinical approach.

This investigation elucidated substantial disparities in prognosis among GC patients based on the site of metastases. Notably, patients with bones metastases exhibited the most ominous prognosis, contrasting with those harboring liver metastases who experienced relatively better outcomes. These findings, in consonance with prior research,^[16]^ accentuate the pivotal role that metastatic sites play in delineating the prognosis of GC patients. It is noteworthy that bones metastases are frequently underdiagnosed, a nuance underscored by autopsy analyses revealing a prevalence of bone metastasis in GC ranging from 13.4% to 15.9%^[17]^, figures significantly higher than the clinical reports of around 4%.^[11,12]^ This incongruity suggests that a considerable number of patients with bones metastases may remain asymptomatic. Definite diagnostic examinations are recommended only when clear clinical symptoms of bone metastasis, such as bone pain, manifest. Consequently, it becomes imperative to identify the risk factors linked to bones metastases in GC and enhance the management of key populations, aiming to improve the prognosis for patients in advanced stages of GC.

In our comprehensive multivariate logistic regression analysis, we unearthed a notable correlation between poorly differentiated (Grade III) GC and an elevated risk of bone metastasis. The heightened invasiveness and migratory capabilities inherent to poorly differentiated tumor cells likely contribute to the propensity for bone metastasis. Our analysis also revealed that Black and Hispanic/Latino patients had a relatively low risk of bone metastasis. One study showed that a higher proportion of NH‐Blacks and Hispanics presented with de novo metastasis compared with NH‐Whites for breast, colorectal, and prostate cancer patients in US.^[18]^ The racial disparity observed in the risk of bone metastasis underscores the role of genetic backgrounds and tumor microenvironments. Gina Kim et al discerned significant variations in the tumor microenvironment between Black and White individuals, a factor intricately linked to the risk of distant recurrence in breast cancer.^[19]^ Additionally, our analysis brought forth an age-related nuance in the risk of bone metastasis. Patients over 50, particularly those exceeding 80 years, exhibited a relatively diminished susceptibility to bone metastasis. This observation resonates with the findings of Rona et al, who reported that the proportion of poorly differentiated GC in the young age group (95.9%) decreased with advancing age.^[20]^ The conjecture here is that the relatively favorable pathological grading of GC in elderly patients may curtail the invasive potential of tumor cells, consequently reducing the risk of bone metastasis with age. This intricate interplay between pathological grading, age, and the likelihood of bone metastasis warrants further exploration and validation in prospective studies.

A striking observation in our analysis is the reversal of risk factors between liver and bones metastases in GC. In contrast to bones metastases, a poorly differentiated (Grade III) pathological grade emerged as a protective factor against liver metastases. The protective influence of the Black race on bones metastases, while heightening the risk of liver metastasis, introduces a nuanced interplay of genetic factors in metastatic patterns. Moreover, unlike bones metastases, the risk of liver metastases escalated in patients over the age of 50, particularly in those exceeding 80 years. The metastasis of GC is a multistep process involving many cytokines, growth factors, chemokines and signaling pathways.^[21,22]^ This process is further complicated by the interaction between tumor cells and the specific tumor microenvironment of host organs.^[23]^ As tumor cells escape the primary malignant tissue via systemic circulation, metastasis occurs,^[24]^ necessitating a deeper exploration of the mechanisms underlying metastasis to different organs in GC.

Our study not only identified several risk factors associated with GC metastases but also pinpointed crucial prognostic factors through Cox regression analyses. In order to enhance clinical utility, we devised a nomogram based on GC metastases. Its accuracy, evaluated through identification and calibration methods, and decision curve analysis, underscores its robust clinical utility. While prior studies have constructed numerous prognostic models for GC,^[25–27]^ the scarcity of models specifically tailored for advanced GC is notable. Furthermore, many existing prognostic models are anchored in molecular signatures derived from gene expression profiles, which, while potent, might pose challenges in clinical application. In contrast, our prognostic model, rooted in the clinicopathological features of patients, is simple, convenient, and holds promise for refining the clinical management of advanced GC.

GC is a highly heterogeneous malignancy, with substantial variations in pathological classification, metastatic patterns, and prognostic outcomes, consistent with our study’s core observations (e.g., liver metastases as the most common at 40.5%, bone metastases with the poorest prognosis, HR = 1.99). Beyond genetic and epigenetic drivers of GC heterogeneity, accumulating evidence highlights key roles of tumor microenvironment heterogeneity. Yasuda and Wang systematically elaborated on this in their review,^[28]^ emphasizing that the GC microenvironment is not a uniform immunosuppressive niche but a dynamically heterogeneous ecosystem. Meanwhile, a study demonstrated the value of immune cell-associated molecular heterogeneity, constructing a B-cell-associated miRNA signature for effective GC prognosis prediction.^[29]^

In the context of our study’s findings, such as the predominance of liver metastases and the poor prognosis of bone metastases, this microenvironment heterogeneity may provide a mechanistic explanation. Recognizing this link between microenvironment heterogeneity and GC metastatic characteristics not only enriches our understanding of the biological basis of organ-specific metastases but also underscores the need for future research to integrate microenvironment-related factors into prognostic models to further refine personalized treatment strategies for advanced GC.

This study, however, carries certain limitations that warrant acknowledgment. Primarily, it adopted a retrospective design, and despite its considerable sample size, potential selection biases may linger. Furthermore, the nomogram devised here hinges on clinical-pathological features. While this choice prioritizes practical application in clinical settings, the omission of tumor molecular features could temper its predictive accuracy. Lastly, the prognostic model for GC unveiled in this study necessitates additional validation through prospective studies to establish its robustness and reliability in diverse clinical scenarios.

In summary, our study offered intricate insights into the metastatic patterns of GC, shedding light on associated risk factors for organ-specific metastases. Moreover, we illuminated the substantial impact of metastatic sites on the prognosis of GC patients. These findings may enhance our comprehension of the metastatic characteristics in GC and furnish a pragmatic clinical prediction model for prognostication in advanced GC patients. However, further validation studies are imperative to solidify the reliability and generalizability of our findings. Additionally, future investigations should delve into potential therapeutic strategies tailored to specific metastatic sites in GC, opening avenues for more effective interventions.

## 5. Conclusion

Our study delved into the metastatic patterns of GC, unraveling associated risk factors for organ-specific metastases. We also underscored the significant impact of metastatic sites on the prognosis of GC patients. These findings contribute to a deeper understanding of the metastatic characteristics in GC and provide a practical clinical prediction model for prognostication in advanced GC patients. Future investigations should focus on validating our findings and exploring therapeutic strategies tailored to specific metastatic sites in GC, paving the way for more effective interventions.

## Author contributions

**Conceptualization:** Jing Wang, Zhimin Wang, Xianglong Tian.

**Data curation:** Guihua Liu, Junjun Gu, Jing Wang.

**Formal analysis:** Guihua Liu, Junjun Gu.

**Investigation:** Junjun Gu, Fang Li.

**Methodology:** Guihua Liu, Junjun Gu, Jing Wang.

**Supervision:** Yi Shen, Zhimin Wang, Xianglong Tian.

**Writing – original draft:** Guihua Liu, Xianglong Tian.

**Writing – review & editing:** Yi Shen, Zhimin Wang, Xianglong Tian.

## Supplementary Material




