# Supplementary material for: Prognostic implications of organ-specific metastases in advanced gastric cancer: A retrospective observational analysis of the SEER database
Source: Medicine (Baltimore). 2025 Oct 31;104(44):e45570. doi: 10.1097/MD.0000000000045570 (PMC12582750; doi:10.1097/MD.0000000000045570)
Supplement: Supplementary file 2 [file medi-104-e45570-s002.pdf]

Variable		N	Odds ratio		P value
Sex	Female	3602		Reference	
	Male	6681		1.52 (1.39, 1.66)	<0.001
Age	less than 50 years	1467		Reference	
	50-64 years	3420		1.52 (1.33, 1.75)	<0.001
	65-79 years	3728		1.72 (1.50, 1.99)	<0.001
	older than 80 years	1668		2.07 (1.76, 2.44)	<0.001
Race	White	7540		Reference	
	American Indian/Alaska Native	87		1.00 (0.64, 1.56)	0.99
	Asian or Pacific Islander	1286		0.76 (0.66, 0.86)	<0.001
	Black	1370		1.35 (1.19, 1.53)	<0.001
Ethnic of orgin	Non Spanish Hispanic Latino	8237		Reference	
	Spanish Hispanic Latino	2046		0.72 (0.64, 0.81)	<0.001
Pathological grading	Well differentiated; Grade I	215		Reference	
	Moderately differentiated; Grade II	1860		1.14 (0.86, 1.52)	0.36
	Poorly differentiated; Grade III	5599		0.56 (0.43, 0.74)	<0.001
	Undifferentiated; anaplastic; Grade IV	212		0.81 (0.55, 1.19)	0.29
	Unknown	2397		0.76 (0.57, 1.01)	0.06
Marry status	Married (including common law)	6175		Reference	
	Seperated or divorced	1085		1.06 (0.93, 1.22)	0.38
	Single (never married)	1757		1.03 (0.92, 1.16)	0.61
	Widowed	1266		1.14 (0.99, 1.30)	0.07
High school education (2008-12)#	Q1	1875		Reference	
	Q2	2137		1.06 (0.93, 1.21)	0.37
	Q3	2502		1.06 (0.93, 1.20)	0.39
	Q4	3769		1.03 (0.89, 1.19)	0.66
Median Household income#	Q1	2633		Reference	
	Q2	2636		1.07 (0.95, 1.20)	0.30
	Q3	2438		1.05 (0.93, 1.19)	0.44
	Q4	2576		1.11 (0.97, 1.27)	0.13

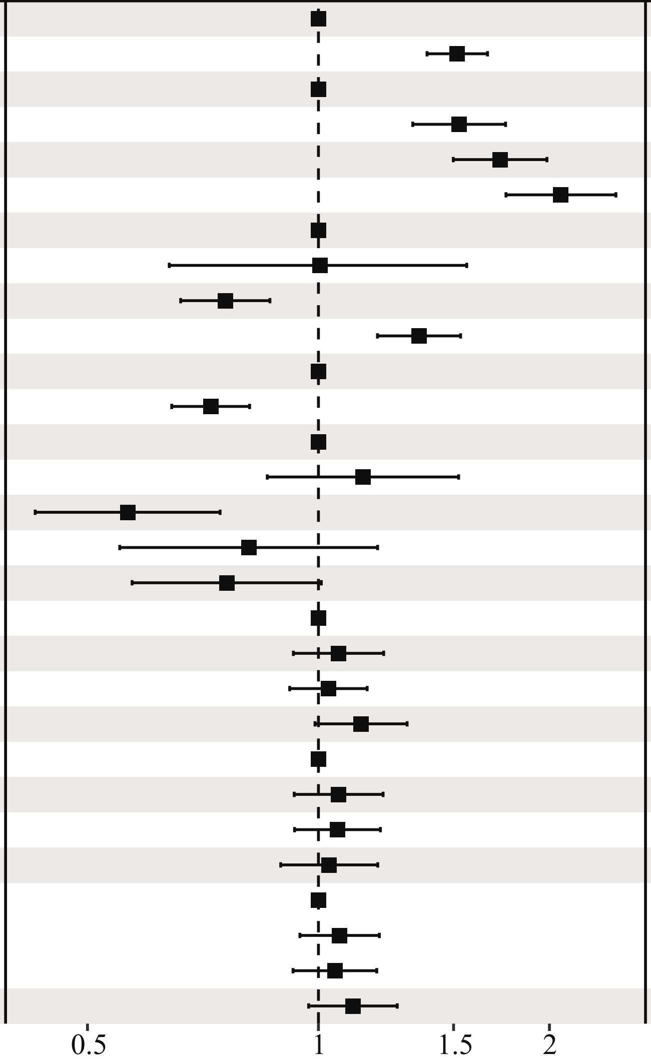

Fig S1: Identification of the risk factors associated with liver metastasis in advanced GC.
Forest plot of Multivariable Logistic analyses of the risk of liver metastasis in GC patients adjusted for sex, age, race, ethnic origin, pathological grading, marital status, education level, and economic income. The black squares on the transverse lines represent the hazard ratio (HR), and the transverse lines represent 95% confidence intervals.
Measure of educational level or economic income for each patient's area of residence is from 2012 American Community Survey data, and it is categorized into equally proportioned quartiles.

Variable		N	Odds ratio	P value
Sex	Female	3602	Reference	
	Male	6681	1.23 (1.08, 1.40)	0.001
Age	less than 50 years	1467	Reference	
	50-64 years	3420	1.03 (0.85, 1.26)	0.750
	65-79 years	3728	1.16 (0.96, 1.41)	0.136
	older than 80 years	1668	1.12 (0.89, 1.41)	0.333
Race	White	7540	Reference	
	American Indian/Alaska Native	87	1.30 (0.72, 2.20)	0.350
	Asian or Pacific Islander	1286	0.77 (0.63, 0.93)	0.007
	Black	1370	0.76 (0.63, 0.91)	0.004
Ethnic of orgin	Non Spanish Hispanic Latino	8237	Reference	
	Spanish Hispanic Latino	2046	0.77 (0.65, 0.90)	0.002
Pathological grading	Well differentiated; Grade I	215	Reference	
	Moderately differentiated; Grade II	1860	0.91 (0.63, 1.34)	0.617
	Poorly differentiated; Grade III	5599	0.67 (0.48, 0.98)	0.032
	Undifferentiated; anaplastic; Grade IV	212	0.43 (0.23, 0.78)	0.006
	Unknown	2397	0.82 (0.57, 1.20)	0.288
Marry status	Married (including common law)	6175	Reference	
	Seperated or divorced	1085	1.07 (0.89, 1.30)	0.458
	Single (never married)	1757	1.12 (0.95, 1.32)	0.169
	Widowed	1266	1.33 (1.10, 1.60)	0.003
High school education (2008-12)#	Q1	1875	Reference	
	Q2	2137	0.97 (0.81, 1.17)	0.754
	Q3	2502	1.07 (0.90, 1.28)	0.441
	Q4	3769	0.96 (0.78, 1.17)	0.678
Median Household income#	Q1	2633	Reference	
	Q2	2636	1.09 (0.93, 1.29)	0.292
	Q3	2438	1.02 (0.86, 1.22)	0.796
	Q4	2576	1.01 (0.84, 1.21)	0.944

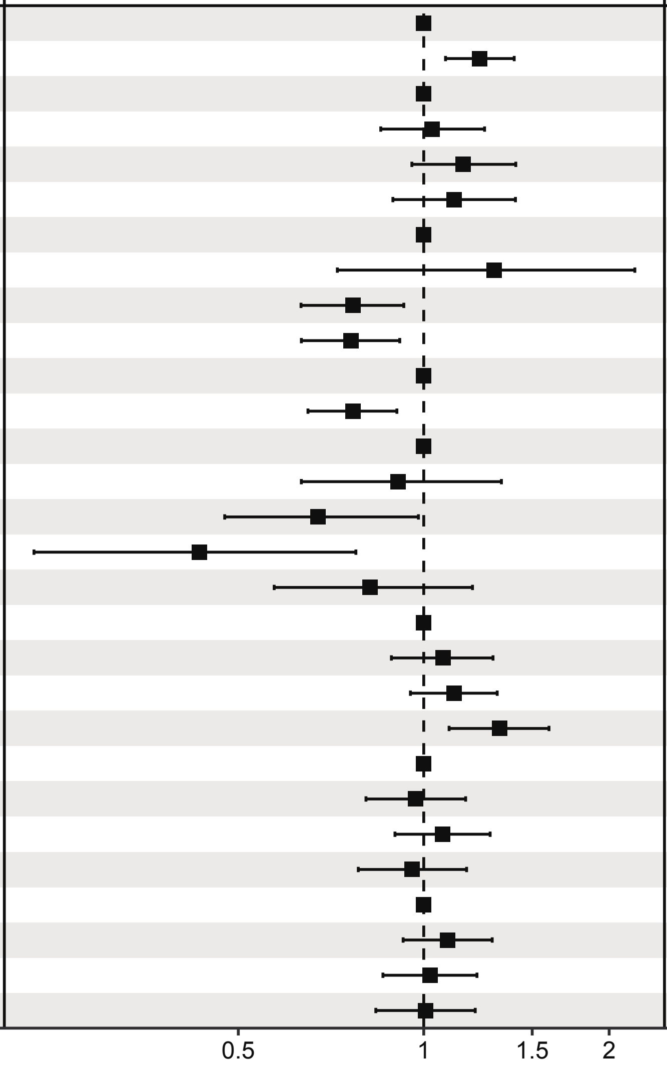

Fig S2: Identification of the risk factors associated with lungs metastasis in advanced GC.

Forest plot of Multivariable Logistic analyses of the risk of lungs metastasis in GC patients adjusted for sex, age, race, ethnic origin, pathological grading, marital status, education level, and economic income. The black squares on the transverse lines represent the hazard ratio (HR), and the transverse lines represent 95% confidence intervals.

Measure of educational level or economic income for each patient's area of residence is from 2012 American Community Survey data, and it is categorized into equally proportioned quartiles.

Variable		N	Odds ratio	P value
Sex	Female	3493	Reference	
	Male	6490	1.34 (0.95, 1.92)	0.100
Age	less than 50 years	1414	Reference	
	50-64 year	3330	0.98 (0.63, 1.57)	0.940
	65-79 years	3623	0.73 (0.46, 1.20)	0.201
	older than 80 years	1616	0.37 (0.18, 0.72)	0.004
Race	White	7387	Reference	
	Asian or Pacific Islander	1261	0.62 (0.35, 1.03)	0.082
	Black	1335	0.47 (0.27, 0.79)	0.007
Ethnic of orgin	Non Spanish Hispanic Latino	7973	Reference	
	Spanish Hispanic Latino	2010	0.60 (0.38, 0.93)	0.028
Pathological grading	Moderately differentiated; Grade II	1842	Reference	
	Poorly differentiated; Grade III	5563	0.60 (0.41, 0.89)	0.009
	Undifferentiated; anaplastic; Grade IV	210	1.01 (0.35, 2.36)	0.982
	Unknown	2368	1.06 (0.71, 1.60)	0.783
Marry status	Married (including common law)	6004	Reference	
	Seperated or divorced	1050	0.99 (0.59, 1.57)	0.963
	Single (never married)	1707	1.12 (0.74, 1.65)	0.589
	Widowed	1222	0.87 (0.45, 1.56)	0.650
High school education (2008-12)#	Q1	1799	Reference	
	Q2	2086	1.22 (0.76, 1.98)	0.406
	Q3	2433	1.15 (0.72, 1.85)	0.568
	Q4	3665	0.97 (0.57, 1.67)	0.918
Median Household income#	Q1	2547	Reference	
	Q2	2574	0.96 (0.62, 1.48)	0.859
	Q3	2377	0.93 (0.60, 1.45)	0.751
	Q4	2485	0.88 (0.55, 1.42)	0.599

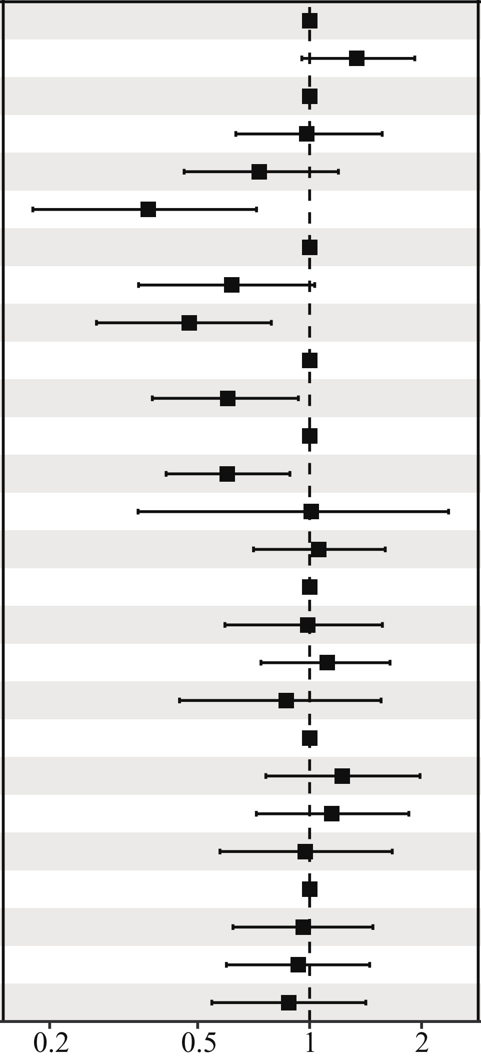

Fig S3: Identification of the risk factors associated with brain metastasis in advanced GC.

Forest plot of Multivariable Logistic analyses of the risk of brain metastasis in GC patients adjusted for sex, age, race, ethnic origin, pathological grading, marital status, education level, and economic income. The black squares on the transverse lines represent the hazard ratio (HR), and the transverse lines represent 95% confidence intervals.

Measure of educational level or economic income for each patient's area of residence is from 2012 American Community Survey data, and it is categorized into equally proportioned quartiles.

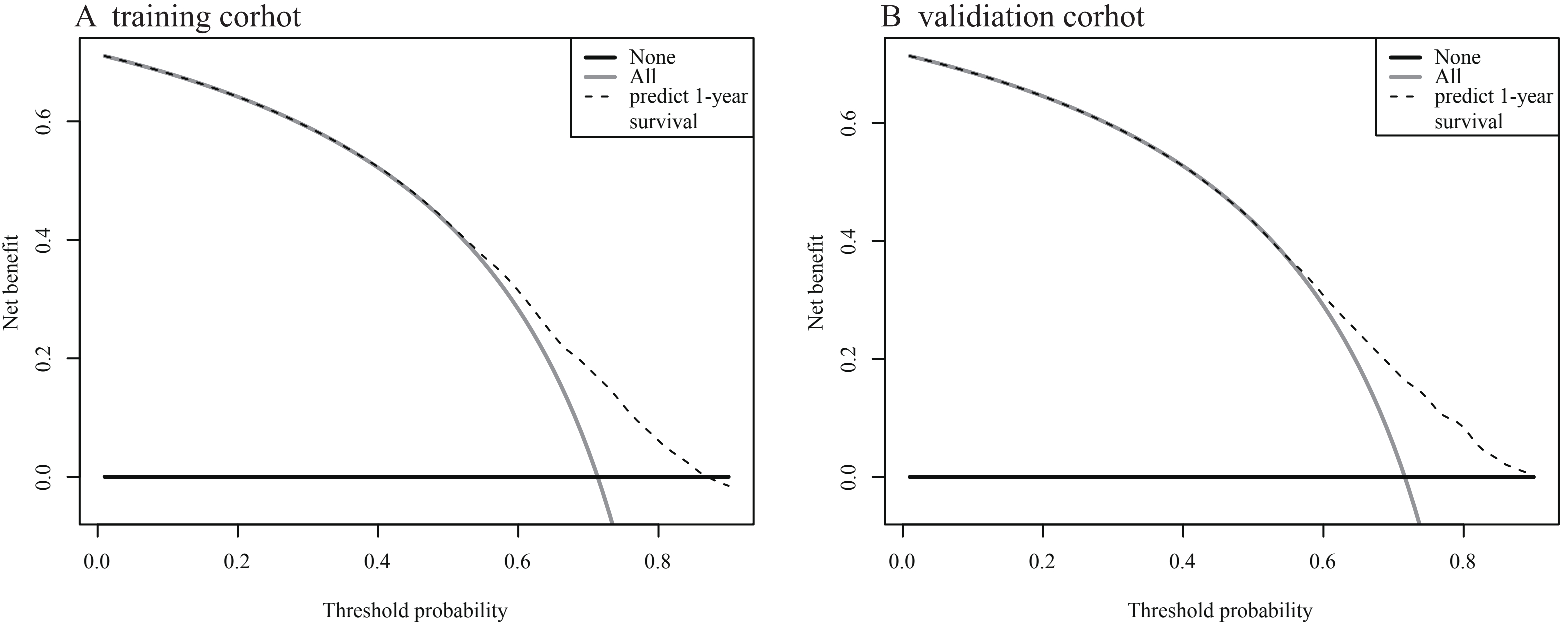

Fig S4: Decision curve analysis (DCA) for assessment of the clinical utility of the nomogram. The analysis results in the training cohort (A) and the validation cohort (B). The x-axis represents the percentage of threshold probability, and the y-axis represents the net benefit.
